# A Comparative Analysis between Enhanced Recovery after Surgery and Traditional Care in the Management of Obstructive Colorectal Cancer

**DOI:** 10.3390/medicina60081319

**Published:** 2024-08-15

**Authors:** Alexandra-Ana Mihăilescu, Minodora Onisâi, Adrian Alexandru, Matei Teodorescu, Cătălin Aliuș, Corneliu-Dan Blendea, Ștefan-Ilie Neagu, Dragoș Șerban, Sebastian Grădinaru

**Affiliations:** 1 Faculty of Medicine, “Carol Davila” University of Medicine and Pharmacy, 050474 Bucharest, Romania; mihailescu.alexandra@drd.umfcd.ro (A.-A.M.); matei.teodorescu@umfcd.ro (M.T.); catalin.alius@umfcd.ro (C.A.); stefan.neagu@umfcd.ro (Ș.-I.N.); dragos.serban@umfcd.ro (D.Ș.); 2 Department of Anesthesiology and Intensive Care Unit, Foisor Hospital Bucharest, 030167 Bucharest, Romania; 3 Hematology Department, Emergency University Hospital Bucharest, 050098 Bucharest, Romania; 4 Department of Plastic and Reconstructive Surgery, Emergency University Hospital Bucharest, 050098 Bucharest, Romania; 5 Neurological Recovery Clinic, Elias University Emergency Hospital, 011461 Bucharest, Romania; 6 4th Surgery Department, Emergency University Hospital Bucharest, 050098 Bucharest, Romania; 7 Faculty of Medicine, Titu Maiorescu University, 040441 Bucharest, Romania; dan.blendea@prof.utm.ro (C.-D.B.); sebastian.gradinaru@prof.utm.ro (S.G.); 8 Department of Recovery, Physical Medicine and Balneology, Ilfov County Emergency Clinical Hospital, 022104 Bucharest, Romania; 9 Department of General Surgery, Ilfov County Emergency Clinical Hospital, 022104 Bucharest, Romania

**Keywords:** colorectal surgery, intestinal obstruction, perioperative management, enhanced recovery

## Abstract

Enhanced Recovery After Surgery (ERAS) represents evidence-based transformation in perioperative care, which has been demonstrated to reduce both recovery times and postoperative complication rates. The aim of the present study was to evaluate the clinical significance of the ERAS program in comparison with conventional postoperative care. This longitudinal cohort observational study enrolled 120 consecutive patients diagnosed with intestinal obstruction caused by colorectal cancers, with 40 patients in the ERAS group and 80 patients receiving conventional postoperative care forming the non-ERAS group. Our study compares the effectiveness of ERAS protocols to non-ERAS methods, focusing on the time to first flatus, defecation, the resumption of normal diet, and early mobilization. The main endpoints are morbidity and hospitalization length. The results showed that despite a longer admission-to-surgery interval in the ERAS group, median hospitalization was significantly shorter compared to the non-ERAS group (*p* = 0.0002). The ERAS group showed a tendency towards a lower incidence of overall postoperative complications, indicating that implementing the ERAS protocol does not increase the risk of postoperative complications, ensuring the safety of enhanced recovery strategies for patients. Also, ERAS patients had notably fewer stomas than those in the non-ERAS group, indicating the potential effectiveness of reducing stoma necessity. This study shows that ERAS surpasses conventional care for colonic or rectal surgery patients, reducing hospital stays and costs while enhancing recovery. This highlights the comprehensive advantages of adopting ERAS in surgical settings.

## 1. Introduction

Colorectal cancer (CRC) represents a major cause of morbidity and mortality worldwide, affecting 1 in 20 people in developed countries, and is the second-leading cause of cancer death, after lung cancer, in both sexes in Europe. Consequently, colon cancer constitutes a public health issue in most European countries. In our country, the incidence of colorectal cancer has doubled in the last 25 years [1,2,3,4].

In the context of treating intestinal blockages caused by colorectal cancer, it is widely recognized that this typically presents as a delayed or postponed emergency. Various methods are employed to manage this condition, such as colon stenting, proximal diversion, and tumor resection. The delay is attributed to the necessity of establishing a preoperative etiological diagnosis and initiating general treatment for hydro-electrolyte balance. On the other hand, delaying surgical intervention after fulfilling these conditions may aim to establish a therapeutic plan where intestinal obstruction and its cause, colorectal tumor, are addressed concurrently or sequentially [5,6,7,8]. This approach enables the implementation of ERAS (Enhanced Recovery After Surgery) principles, aiming for reduced operative stress and enhanced patient recovery. The goal is to achieve quicker discharge from the hospital and faster resumption of daily activities following major or minor surgeries, all while maintaining low rates of complications and readmissions. Implementing the ERAS program for colorectal surgery consistently results in a reduction in postoperative complications, expedited patient recovery, a decrease in hospitalization duration, and reduced healthcare expenses [9,10,11,12,13,14].

ERAS, also known as fast-track surgery or multimodal surgery, uses a range of techniques to improve surgical conditions and recovery processes. The aim is to ensure patients are discharged from the hospital sooner and resume their normal activities more quickly after both major and minor surgeries without raising the risk of complications or the need for readmissions. For instance, within the ERAS program, managing postoperative pain without opioid analgesia is preferred due to the negative impact of opioids on respiratory function, the central nervous system, and gastrointestinal function [15,16,17]. After surgery, prolonged bed rest is not recommended as it can hinder pulmonary function, reduce tissue oxygenation, and increase the risk of pulmonary complications. To prevent these issues, initiating patient mobilization as early as possible is crucial for enhancing postoperative care [17,18,19,20,21,22,23].

The objective of this study was to compare the mortality and morbidity of patients undergoing colonic or rectal resection following either the ERAS program or conventional postoperative care. This evaluation aims to determine the clinical implications of enhanced perioperative management.

## 2. Materials and Methods

### 2.1. Identification of Patients

During the 36-month study period, 162 patients were hospitalized for colorectal cancer with clinically and radiologically proven intestinal obstruction (abdomen and pelvis computed tomography with intravenous contrast). In total, 24 patients underwent colonic stent insertion (for palliative care or as a bridge to surgery), 30 patients had a diversion stoma installed in continuity, and the remaining 120 cases underwent emergency tumor resection.

We performed a longitudinal cohort observational prospective study. We included all the patients (*n* = 120) who were diagnosed with intestinal obstruction due to colorectal cancers treated between January 2018 and December 2021 at the Emergency University Hospital in the Department of Surgery IV, Bucharest, Romania. 

Written informed consent was obtained from all patients. Patients were treated according to care routines considered traditional at that time (traditional group, *n* = 80) and according to a modified and adapted ERAS protocol for patients with obstructive colorectal cancer (ERAS group, *n* = 40). Since then, the ERAS protocol has become the standard of care for all patients undergoing elective colorectal resection in our department. The same two surgeons who cared for the patients in both study periods utilized the same ERAS care principles. 

The nutritional status was classified using serum albumin levels in both groups. Nutritional supplements were administered preoperatively to patients with malnutrition and/or preoperatively to improve symptomatic obstruction. Evidence of complete or near-complete colonic obstruction was confirmed by computed tomography (CT). 

### 2.2. Criteria of Exclusion

Patients with clinical peritonitis, those with recurrent obstructive tumors, and those receiving neoadjuvant treatments were excluded. Non-resectable and metastatic patients were also excluded.

### 2.3. ERAS Protocol

Some elements of the ERAS protocol (such as intraoperative epidural analgesia and avoidance of routine postoperative abdominal drainage and nasogastric tubes) were already routine practices at the beginning of the study and, consequently, were part of the traditional perioperative care for elective patients. The summary of major differences in perioperative protocols between the ERAS program and conventional care management is presented in Table 1.

All clinical outcomes were recorded prospectively. Nutrition, dietary intake, fluid balance, and mobilization were recorded daily until discharge. The degree of mobilization was jointly documented by both medical staff and patients in a patient journal. Identical discharge criteria were used throughout the study period (the patient must be fully mobilized, afebrile, tolerating normal or near-normal amounts of solid and liquid foods, have restored bowel function, and have adequately controlled pain without opioids). Other variables taken into consideration were the following:

- ASA (American Society of Anesthesiologists) score—a classification system used to assess and communicate a patient’s preoperative health status. The ASA score helps predict perioperative risks by categorizing patients based on their overall health and comorbidities;
- Duration of obstruction (days)—the time without gastrointestinal transit at the time of admission;
- Estimated blood loss (mL)—the assessment of intraoperative blood loss;
- Locoregional anesthesia—includes the placement of high thoracic or lumbar epidural catheters, subfascial blocks, and local anesthesia;
- Intra/postoperative IV (mL)—the volume of fluids administered intra/postoperatively;
- Time to first flatus (days)—the time until the patient first passes gas, indicating the return of gastrointestinal function;
- Time to first defecation (days)—the time until the patient first has a bowel movement, signifying the resumption of normal bowel function;
- Postoperative complications—monitored as separate variables without discovering a statistically significant difference between the two groups, which is why they were discussed generically. Complications were diagnosed within the first 30 postoperative days according to predefined criteria. Surgical staff, technical aspects of the surgery, as well as the choice of antibiotics and thromboprophylaxis, remained unchanged during the study period.

### 2.4. Statistical Analysis

All data were prepared and compiled using SPSS software (version 15.0 for Windows). Means and standard deviations or medians (ranges) are presented for continuous data. Unpaired *t*-tests were used to compare data between the two groups when they showed a normal distribution. Mann–Whitney U tests were used when data were not normally distributed. Pearson/Spearman tests or Fisher’s exact tests were used for categorical data. A *p* value less than 0.05 was considered statistically significant. Unless otherwise specified, comparisons are between the traditional and ERAS groups.

## 3. Results

Of the 120 patients who underwent tumor excision during the same hospitalization period, 40 patients (33.3%) were cared for through the ERAS program. For comparison, 80 patients receiving conventional postoperative care during the study period were included in the non-ERAS group. The two groups were comparable in terms of age, gender, BMI (body mass index), ASA (American Society of Anesthesiologists) grade, preoperative hemoglobin level, and preoperative serum albumin level. The characteristics and operative details of ERAS and non-ERAS patients are presented in Table 2. It is noteworthy that no diversion ileostomy was performed in cases of tumor resection with primary anastomosis.

The ASA score was similar for both the ERAS and non-ERAS groups. This similarity in ASA scores indicates that the patients in both groups had comparable baseline health statuses and surgical risk profiles, allowing for a more accurate comparison of the outcomes between the two protocols. The homogeneity of the study population enhances the reliability of the findings, suggesting that any observed differences in outcomes can be attributed more confidently to the ERAS protocol rather than to variations in patient health conditions.

Patients were stratified based on the type of surgery: colonic resection (with or without anastomosis) or colorectal resection (under peritoneal reflection for tumors of the upper rectum and recto-sigmoid junction). All data were recorded, including patient demographics, operative details (tumor location, type of procedure, operative time, and estimated blood loss), pathological staging, and postoperative outcomes. Table 3 presents data regarding surgical procedures. Postoperative outcomes included postoperative complications (infectious, respiratory, hemorrhagic, fistulas, eviscerations), time to first flatus, time to first defecation, time to resume normal diet, length of hospital stay, hospital readmission, and mortality. All patients were scheduled for follow-up at 30 days postoperatively. Data regarding operative and postoperative details are presented in Table 4. Adjuvant chemotherapy is commonly recommended after the curative resection of obstructed colorectal cancer. However, the decision to initiate adjuvant treatment depends on the agreement between patients and oncologists. The type of analgesia administered is presented in Table 5.

Patients in the ERAS group had significantly fewer stomas compared to patients in the non-ERAS group. This suggests that the ERAS protocol may effectively reduce the need for stomas.

The median hospitalization duration was significantly shorter in the ERAS group compared to the non-ERAS group [6.8 days (range: 4–12) vs. 10.7 days (range: 6–32), *p* = 0.0002], despite a significantly longer interval between admission and surgery in the ERAS group. There was a stronger and statistically significant correlation between the admission-to-surgery interval and the duration of postoperative hospitalization in the ERAS group (0.658 and *p* = 0.002) compared to the non-ERAS group (0.339 and *p* = 0.032).

The incidence of overall postoperative complications tended to be lower in the ERAS group (50% vs. 60%), but this difference did not reach statistical significance (*p* = 0.238). However, in the ERAS group, the risk of postoperative complications was 4.846 times higher in patients undergoing open surgery compared to those who underwent laparoscopic surgery (69.23% vs. 14.29%, *p* = 0.015). There were 2 deaths in the non-ERAS group (2.5%), 6 reoperations, and a total of 12 readmissions in both groups. The deaths were due to thrombotic (extensive acute mesenteric ischemia) and TEP (pulmonary thromboembolism) accidents, respectively. Two additional reoperations were required for non-ERAS patients for anastomotic fistulas, and they were eventually discharged in good condition but after prolonged hospitalizations. In the ERAS cohort, there were two readmissions for perianastomotic pelvic abscesses that required laparoscopic reintervention for drainage, with favorable outcomes leading to the spontaneous closure of the fistula. The other four readmissions in the ERAS group were resolved without surgery in the operating room, including a blocked evisceration, revision of the colostomy for minimal bleeding, wound hematoma, and C. diff. infection. There was only one day of readmission in the ICU (Intensive Care Unit) for patients in the ERAS group and thirty-three days for patients in the non-ERAS group, which was the main reason for the significant cost differences between the two groups.

**Figure 1:**
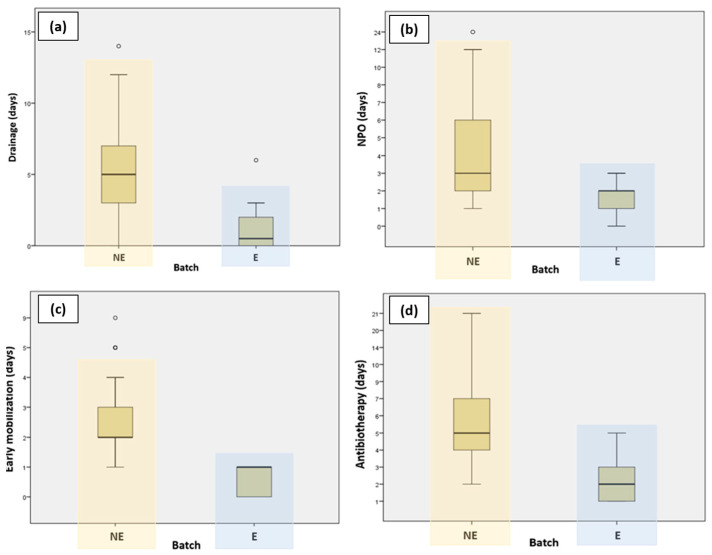
Graphic representation comparing ERAS and non-ERAS groups in terms of (**a**) duration of drainage; (**b**) NPO (nil per os); (**c**) early mobilization; and (**d**) antibiotherapy.

Patients who followed the ERAS protocol had their drain tubes removed much faster compared to those who did not follow the ERAS protocol. Figure 1 presents a graphic representation comparing the different variables for the ERAS and non-ERAS groups.

Patients who were part of the ERAS protocol experienced a significantly shorter length of hospital stay compared to those who were not part of the ERAS program. This decrease in hospitalization days highlights the effectiveness of the ERAS protocol, which involves comprehensive perioperative care strategies designed to accelerate recovery and reduce postoperative complications. The findings demonstrate the potential of ERAS protocols to improve patient outcomes by facilitating quicker recoveries and decreasing the overall burden on healthcare resources. 

Goal-directed fluid therapy (GDFT) aims to maintain adequate organ perfusion to deliver sufficient oxygen to all organs during and after surgery. The key components involve optimizing flow by maintaining the patient’s vascular volume while avoiding the harmful effects of hypotension. In daily practice, several hemodynamic variables, such as heart rate, mean arterial pressure, and central venous pressure, are measured to adjust the amount of intravenous fluid, blood transfusion, and vasopressor administration. In a GDFT protocol, advanced monitoring devices, such as transesophageal Doppler monitoring or a non-invasive cardiac output monitor, may be required.

**Figure 2:**
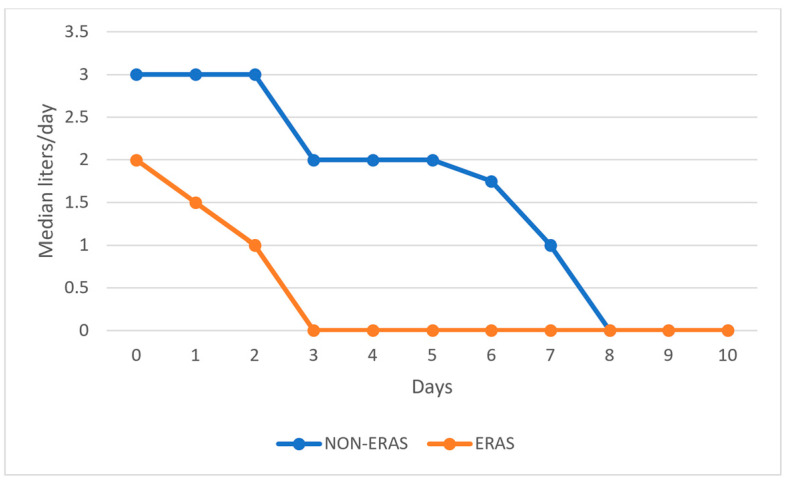
Graphical representation of the median volume of fluids administered postoperatively by day.

Patients in the ERAS group received significantly fewer fluids postoperatively compared to those in the non-ERAS group, as shown in Figure 2.

There were no significant differences in the complication rates as well as readmission rates between the ERAS group and the non-ERAS group. However, the ERAS group had somewhat fewer complications compared to the non-ERAS group. This suggests that the implementation of the ERAS protocol does not increase the risk of postoperative complications, providing reassurance that these enhanced recovery strategies are safe for patients. The findings indicate that while the ERAS protocol is designed to improve recovery times and reduce hospital stays, it does so without negatively impacting the overall complication rates. This balance of improved efficiency while maintaining safety highlights the potential benefits of incorporating ERAS protocols into standard surgical practices.

Patients in the ERAS group had a shorter length of hospital stay compared to those in the non-ERAS group. This reduction in hospitalization days highlights the effectiveness of the ERAS protocol in enhancing postoperative recovery.

In the ERAS protocol, the patient’s recovery is more predictable, which allows for a more accurate estimation of the discharge date. This predictability stems from the implementation of standardized, evidence-based procedures that reduce variability in postoperative recovery. In both elective and emergency surgery, shortening the length of hospital stay is the primary objective achieved.

One of the key components of ERAS protocols is the early resumption of oral intake. ERAS guidelines often recommend starting clear liquids a few hours after surgery and advancing to solid foods as tolerated, usually within 24 h postoperatively. By minimizing the duration of NPO (nil per os), ERAS aims to enhance gastrointestinal recovery, reduce the risk of postoperative ileus (intestinal blockage), and improve overall patient outcomes. 

Early mobilization is a crucial component of postoperative care and differs significantly between ERAS protocols and traditional (non-ERAS) care. Patients in the ERAS group were encouraged to get out of bed and start moving as soon as possible after surgery (up to 24 h postoperatively). Early mobilization helps reduce the risk of complications, such as deep vein thrombosis (DVT), pulmonary embolism, and pneumonia. It promotes quicker return-to-normal activities, enhances muscle strength, and improves overall functional recovery. Patients who mobilize early typically have shorter hospital stays and faster recovery times. 

ERAS protocols typically include the administration of prophylactic antibiotics just before surgery to reduce the risk of surgical site infections (SSIs). ERAS protocols favor a single dose or a very limited course of antibiotics (usually less than 24 h) postoperatively unless there is a specific indication for longer use. In summary, ERAS protocols advocate for the judicious use of antibiotics, with a focus on preoperative prophylaxis and minimal postoperative duration, which reduces the risk of complications and antibiotic resistance.

## 4. Discussion

Several studies revealed that the implementation of an ERAS program in emergency tumor resection for obstructing colorectal cancer resulted in a significantly shorter hospital stay and quicker recovery of bowel function compared to conventional postoperative care, all without an increase in 30-day mortality or readmission rates [24,25,26,27]. This underscores the potential of ERAS protocols to improve outcomes in emergency colorectal surgery cases.

Another study examined the application of the ERAS protocol in patients undergoing emergency major abdominal surgery. The group following the ERAS protocol showed significant reductions in catheter and drain usage, urinary tract infections, urinary retention, patient-controlled analgesia, and chest infections. Although these findings endorse the integration of ERAS in emergency abdominal surgeries, it is noted that only certain ERAS guidelines were followed, indicating the necessity for additional research [28].

In a retrospective cohort study comprising 370 patients undergoing major abdominal surgery, both with and without the implementation of an ERAS protocol, comprehensive data were collected, encompassing admission and operative details, postoperative management, and outcomes. Notably, among the patients following the ERAS protocol, there were significant reductions observed in both intraoperative (*p* < 0.001) and postoperative (*p* < 0.001) intravenous fluid administration. Moreover, a significantly lower proportion of ERAS patients required catheters (*p* < 0.001), drains (*p* = 0.001), and patient-controlled analgesia for more than 2 days (*p* = 0.01). Additionally, both major and minor complications were markedly reduced in patients adhering to the ERAS protocol. These findings underscore a substantial shift towards incorporating ERAS principles in the management of emergency patients following the successful implementation of such programs in elective cases [29].

Numerous studies have shown that in emergency major abdominal surgeries, such as colorectal surgery, the use of minimally invasive techniques has been linked to improved clinical outcomes, including reduced mortality and shorter hospital stays. Additionally, minimally invasive surgery has been associated with lower rates of surgical site infections and decreased lengths of stay compared to conventional approaches [30,31,32,33,34,35,36,37,38].

Our findings revealed that despite a notably longer interval between admission and surgery in the ERAS group, the median hospitalization duration was significantly shorter at 6.8 days (range: 4–12) compared to the non-ERAS group’s median duration of 10.7 days (range: 6–32), showing a statistically significant difference (*p* = 0.0002). This indicates that the implementation of the ERAS protocol may contribute to a more efficient recovery process and reduced hospital stays.

Despite being in the ERAS group, patients who underwent open surgery had a substantially elevated risk of postoperative complications: 4.846 times more likely compared to their counterparts who underwent laparoscopic surgery. The incidence of complications was notably higher in the open surgery subgroup at 69.23%, in stark contrast to the laparoscopic surgery subgroup, which had a significantly lower rate of 14.29%. This observed disparity underscores the importance of the surgical approach in mitigating postoperative complications within the ERAS framework. This highlights the potential benefits of laparoscopic techniques in reducing complications within the ERAS protocol.

The limitation of this study lies in its relatively small sample size, which may affect the generalizability and statistical power of the results. This is partly due to the fact that the ERAS (Enhanced Recovery After Surgery) method has not yet been widely adopted as a recommended protocol, with few surgical teams having experience in its implementation. This is especially relevant since the protocol is adapted for emergency situations. However, the study’s strengths include the consideration of over 50 variables, from which the most relevant and statistically significant ones have been selected and discussed for their medical importance.

## 5. Conclusions

Patients in the ERAS group had fewer stomas and received fewer postoperative fluids compared to those in the non-ERAS group. Additionally, patients following the ERAS protocol had a quicker removal of drain tubes compared to those not following the protocol. However, patients in the ERAS group who underwent open surgery had a significantly higher risk of postoperative complications compared to those who underwent laparoscopic surgery. Complication and readmission rates did not differ significantly between the ERAS group and the non-ERAS group. Nonetheless, the ERAS group experienced slightly fewer complications. Additionally, patients in the ERAS group had shorter hospital stays compared to those in the non-ERAS group.

ERAS guidelines are already commonly used in practice for elective patients. However, there have been precautions regarding the use of ERAS in emergency cases due to a perceived higher risk of complications. Our study has demonstrated that there are no significant differences in the risk of complications between emergency cases managed with ERAS and those managed with traditional methods. This finding supports the successful implementation of ERAS in emergency situations, indicating that it can be applied without imposing additional risks on patients.

The findings of this study indicate that the ERAS program outperforms conventional postoperative care for patients undergoing colonic or rectal surgery. Implementing the ERAS protocol can lead to reduced stomas and potentially fewer complications, although the latter may not be statistically significant compared to traditional care. Additionally, the ERAS protocol has demonstrated significant reductions in hospital stays and costs, all while improving the recovery of patients. This underscores the multifaceted benefits of implementing an ERAS approach in surgical settings.

## Figures and Tables

**Table 1:** Perioperative protocol of the Enhanced Recovery After Surgery protocol and comparison with conventional care.

Perioperative Protocol	Non-ERAS	ERAS
Preoperative patient education and counseling	General counseling and advice provided exclusively by surgeons	Comprehensive guidance and education offered through written brochures by surgeons, nurses, and anesthesiologists
Preoperative fasting	No food and no drink	Oral intake should occur up to 2 h before anesthesia induction
Perioperative fluid management	No prevention of excessive fluid intake	Goal-directed fluid management during surgery, followed by controlled fluid administration in the postoperative period
Intraoperative hypothermia	Sometimes	No. Active body warming
Nasogastric tube	Removed by postoperative day 3	Removed promptly after surgery
Postoperative nonopioid medications	Postoperative pain relief frequently depends on the administration of opioid medications via intravenous delivery	Pain management typically involves the use of nonopioid medications
Postoperative fasting	No oral intake is permitted for three days following surgery.	Consume water two hours post-surgery; transition to oral nutritional supplements on postoperative day 1
Stimulation of gut motility	No	Post-surgery regular laxative (magnesium oxide)
Mobilization care	Standard walking regimen, usually starting on postoperative day 2	Engagement in activities outside of bed on postoperative day 1
Drainage tubes	Removal within 3 days postoperation	Avoided

**Table 2:** Patient characteristics and operative details.

Data	Non-ERAS	ERAS	*p*
N	80	40	
Age	68.3 ± 8.17	66.4 ± 9.88	0.4372
Gender			
Males	44 (55%)	24 (60%)	0.3628
Females	36 (45%)	16 (40%)	
BMI	29.83 ± 4.6	27.05 ± 4.93	0.0478
ASA grades			
II	36 (45%)	18 (45%)	0.9557
III	34 (42.5%)	18 (45%)	
IV	5 (12.5%)	4 (10%)	
Hb	11.575 ± 2.04	10.93 ± 2.01	0.2394
Albumin	3.57 ± 0.47	3.51 ± 0.59	0.7594
Tumor location			
Right/left side	29 (36.25%)/51 (63.75%)	14 (35%)/26 (65%)	0.2343
Ascending colon	13 (16.25%)	7 (17.5%)	0.9415
Hepatic flexure	8 (10%)	4 (10%)	
Transverse colon	8 (10%)	3 (7.5%)	
Splenic flexure	5 (6.25%)	2 (5%)	
Descending colon	10 (12.5%)	6 (15%)	
Sigmoid colon	12 (15%)	5 (12.5%)	
Recto-sigmoid junction	11 (13.75%)	7 (17.5%)	
Superior rectus	13 (16.25%)	6 (15%)	

* Data are presented as numbers and mean ± SD; percentages are presented in parentheses. BMI = body mass index; ASA = American Society of Anesthesiologists; Hb = preoperative hemoglobin level; and albumin = preoperative serum albumin level.

**Table 3:** Surgical procedures.

Surgical Procedure	Non-ERAS (*n* = 80)	ERAS (*n* = 40)	*p*
HD	26 (32.5%)	18 (45%)	0.2657
HS	20 (25%)	8 (20%)	
RRS	22 (27.5%)	14 (35%)	
RS	12 (15%)	0	

HD = right-sided hemicolectomy; HS = left-sided hemicolectomy; RRS = recto-sigmoid resection; and RS = segmental/sigmoid resection. ** Data are presented as numbers; percentages are presented in parentheses.

**Table 4:** Operative and postoperative data.

Data	Non-ERAS *n* = 80	ERAS *n* = 40	*p*
Duration of obstruction (d)	5.95	5.75	0.8742
Operative time (min)	154.12 ± 38.7	175.5 ± 52.11	0.1260
Estimated blood loss (mL)	327.5 ± 221.8	305 ± 216.97	0.5542
Locoregional anesthesia	10 (12.5%)	32 (80%)	**0.0000**
Intraoperative IV	5.08 ± 1.35	2.67 ± 0.97	**0.0000**
Postoperative IV	20.8 ± 9.01	5.15 ± 2.42	**0.0000**
Stoma	54 (79.41%)	7 (20.59%)	**0.0103**
Drainage days	5.45 ± 3.79	1.25 ± 1.61	**0.0000**
Time to first flatus (d)	2.45 ± 2.57	2.5 ± 1.63	0.4791
Time to first defecation (d)	3.90 ± 3.74	3.7 ± 2.31	0.7877
Length of hospital stay (d)	10.75 ± 5.3	6.85 ± 2.39	**0.0002**
Postoperative complications	48 (60%)	20 (50%)	0.2386

d = days; min = minutes. * Data are presented as numbers and mean ± SD; percentages are presented in parentheses.

**Table 5:** Type of analgesia administered.

Analgesia Type	Non-ERAS *n* = 80	ERAS *n* = 40
NSAIDs	9 (11.25)	0
B	0	6 (15%)
EC	10 (12.5%)	12 (30%)
EC + AINS	0	2 (5%)
EC + B	0	8 (20%)
EC + L	0	2 (5%)
L	0	6 (15%)
L + B	0	2 (5%)
OP	34 (42.5%)	2 (5%)
OP + AINS	24 (30%)	0
STD	3 (3.75%)	0

NSAIDs = nonsteroidal anti-inflammatory drugs; B = blocks; EC = epidural catheter; L = lidocaine; OP = opioids; and STD = steroids. * Data are presented as numbers; percentages are presented in parentheses.

## Data Availability

The data presented in this study are available on reasonable request from the corresponding authors.
